# The Utility of Efavirenz-based Prophylaxis Against HIV Infection. A Systems Pharmacological Analysis

**DOI:** 10.3389/fphar.2019.00199

**Published:** 2019-03-13

**Authors:** Sulav Duwal, Daniel Seeler, Laura Dickinson, Saye Khoo, Max von Kleist

**Affiliations:** ^1^Department of Mathematics and Computer Science, Systems Pharmacology and Disease Control, Institute of Bioinformatics, Freie Universität Berlin, Berlin, Germany; ^2^Department of Molecular and Clinical Pharmacology, University of Liverpool, Liverpool, United Kingdom

**Keywords:** PrEP, modeling, PK-PD, translation, repurposing, resource-constrained, cost-efficient, PEP

## Abstract

Pre-exposure prophylaxis (PrEP) is considered one of the five “pillars” by UNAIDS to reduce HIV transmission. Moreover, it is a tool for female self-protection against HIV, making it highly relevant to sub-Saharan regions, where women have the highest infection burden. To date, Truvada is the only medication for PrEP. However, the cost of Truvada limits its uptake in resource-constrained countries. Similarly, several currently investigated, patent-protected compounds may be unaffordable in these regions. We set out to explore the potential of the patent-expired antiviral efavirenz (EFV) as a cost-efficient PrEP alternative. A population pharmacokinetic model utilizing data from the ENCORE1 study was developed. The model was refined for metabolic autoinduction. We then explored EFV cellular uptake mechanisms, finding that it is largely determined by plasma protein binding. Next, we predicted the prophylactic efficacy of various EFV dosing schemes after exposure to HIV using a stochastic simulation framework. We predicted that plasma concentrations of 11, 36, 1287 and 1486ng/mL prevent 90% sexual transmissions with wild type and Y181C, K103N and G190S mutants, respectively. Trough concentrations achieved after 600 mg once daily dosing (median: 2017 ng/mL, 95% CI:445–9830) and after reduced dose (400 mg) efavirenz (median: 1349ng/mL, 95% CI: 297–6553) provided complete protection against wild-type virus and the Y181C mutant, and median trough concentrations provided about 90% protection against the K103N and G190S mutants. As reduced dose EFV has a lower toxicity profile, we predicted the reduction in HIV infection when 400 mg EFV-PrEP was poorly adhered to, when it was taken “on demand” and as post-exposure prophylaxis (PEP). Once daily EFV-PrEP provided 99% protection against wild-type virus, if ≥50% of doses were taken. PrEP “on demand” provided complete protection against wild-type virus and prevented ≥81% infections in the mutants. PEP could prevent >98% infection with susceptible virus when initiated within 24 h after virus exposure and continued for at least 9 days. We predict that 400 mg oral EFV may provide superior protection against wild-type HIV. However, further studies are warranted to evaluate EFV as a cost-efficient alternative to Truvada. Predicted prophylactic concentrations may guide release kinetics of EFV long-acting formulations for clinical trial design.

## 1. Introduction

The ambitious goals formulated by UNAIDS are to end AIDS by 2030 (UNAIDS, 2017). However, unlike many other infections, no cure is available to clear HIV infection. Ending AIDS therefore heavily relies on strategies to reduce the number of new HIV infections from an estimated 2.1 million in 2014 (UNAIDS, 2015) to 500,000 cases by 2020 and to fewer than 200,000 by 2030 (UNAIDS, 2016). While a vaccine would be the ideal tool for the purpose, intrinsic difficulties have so far precluded the development of an effective vaccine against HIV. Despite these setbacks, the development of about 30 antiviral compounds to stop HIV replication has been an overwhelming success (Gulick, 2018) in HIV research.

In light of the current situation, recent years have seen an increasing interest in utilizing antivirals not only for treatment, but also to prevent HIV transmission. Two general strategies are currently investigated for this purpose:

(i) Treatment-as-prevention (TasP) intends to put individuals with an HIV diagnosis immediately on treatment, which essentially makes them non-contagious (Cohen et al., 2011). However, a major limitation of this approach is that HIV is typically transmitted early after infection (Brenner et al., 2007; Yousef et al., 2016), when the recently infected individual is unaware of his/her HIV status and has consequently not initiated TasP. Thus, maximizing the epidemiological impact of TasP also requires to improve HIV diagnosis, which is a central component of the 90-90-90 strategy (UNAIDS, 2017).

(ii) Pre-exposure prophylaxis (PrEP) acts on the viral dynamics in the virologically challenged individual immediately after virus exposure. Akin to a vaccination, PrEP increases the probability that transmitted virus gets cleared, protecting individuals from becoming irreversibly infected. However, unlike vaccination, PrEP protection is a direct function of the concentration of prophylactic drugs at the target site.

Once-daily oral PrEP with the drug combination Truvada (tenofovir disoproxil fumarate-emtricitabine) has been approved since 2012 in the US and since 2016 in the EU. Initial clinical studies with Truvada demonstrated its utility as a PrEP agent (Grant et al., 2010), while subsequent studies indicated that the efficacy of Truvada-based PrEP was highly dependent on the individual's adherence to the once daily regimen. While it is difficult to quantify PrEP adherence clinically (Haberer et al., 2015), efficacy estimates in apparently highly adherent individuals were 86–100% in the IPrEx OLE study, 58–96% in the PROUD study and 96% in the Partners PrEP OLE study (Grant et al., 2014; McCormack et al., 2016). The VOICE and FEM-PrEP studies indicated that Truvada may not prevent infection in poorly adherent individuals, i.e., if 30% of individuals had detectable drug in their blood plasma (Van Damme et al., 2012; Marrazzo et al., 2015). Mathematical modeling of Truvada-based PrEP (Duwal et al., 2016) established the precise relationship between drug pharmacokinetics and prophylactic efficacy confirming many clinical observations (i.e., quantifying the prophylactic efficacy to be ≈96% in fully adherent individuals).

While adherence is a major current concern that motivates the identification of novel long-acting drug candidates and optimized deployment strategies (AVAC, 2019), a currently neglected factor is the cost of PrEP, with the majority of HIV infections occurring in resource-constrained countries (UNAIDS, 2016). Keller and Smith (2011) noted that the price of Truvada currently undermines the advancement of pre-exposure prophylaxis, particular in resource-constrained settings. Yet regrettably, current PrEP research focusses entirely on patent-protected compounds (AVAC, 2019). This makes it unlikely that a current, or next-generation PrEP regimen will become broadly implemented in resource-constrained regions where they could benefit most. Moreover, PrEP is the only strategy by which women can protect themselves against HIV infection, making PrEP highly relevant in regions like sub-Saharan Africa, where young women are the most relevant target group to halt the ongoing spread of HIV (Dellar et al., 2015; Maxmen, 2016), accounting for ≈ 7000 infections per week (Mathur et al., 2016).

A natural progression would therefore be whether currently neglected, patent-expired compounds might make good candidates for PrEP repurposing. Based on an initial computational assessment of potential candidates (Duwal et al., 2019), we focus herein on the patent-expired non-nucleoside reverse transcriptase inhibitor (NNRTI) efavirenz (EFV), which is successfully used in HIV treatment, particularly in resource-constrained settings, where it costs as little as 0.1US*$* per day. To this end, we assess efavirenz pharmacokinetics, consider its mode of action and establish the relationship between pharmacokinetics and prophylactic efficacy. Since reduced-dose (400 mg) efavirenz has a considerably improved safety profile, we assess the prophylactic efficacy of 400 mg oral EFV when used in chronic PrEP, PrEP on demand and post-exposure prophylaxis (PEP).

## 2. Patients

A previously developed population pharmacokinetic (PK) model, constructed using data collected as part of ENCORE 1 was used. ENCORE 1 was a multi-center, double-blind, placebo-controlled trial designed to compare standard dose efavirenz (600 mg once daily) to a reduced dose (400 mg once daily) in HIV-infected, treatment-naive adults. Patients recruited at sites across Africa, Asian, South America, Europe and Oceania were randomized (1:1) to receive efavirenz 600 or 400 mg once daily in combination with tenofovir disoproxil fumarate/emtricitabine (Truvada, 300/200 mg once daily) (ENCORE1 Study Group, 2014; ENCORE1 Study Group et al., 2015).

At weeks 4 and 12 of therapy, single random blood samples were drawn between 8-16 hours post-dose, additionally intensive sampling was undertaken in a subgroup of patients between weeks 4 and 8 [pre-dose (0 h), 2, 4, 8, 12, 16 and 24 h post-dose]. Plasma efavirenz was quantified using a validated HPLC-MS/MS method (Amara et al., 2011). Overall, 606 patients (n=131, 32% female) randomized to efavirenz 600 mg (*n* = 311) and 400 mg once daily (*n* = 295) contributed 1491 samples for model development [median (range) 2 (1–9) per patient]. Median (range) age and weight were 35 years (18–69) and 65kg (39–148) and baseline viral load ranged between 162 and 10,000,000 copies/mL. The majority of patients were of African and Asian ethnicity (37 and 33%, respectively) with the remainder identifying as Hispanic (17%), Caucasian (13%) and Aboriginal and Torres Strait Islander (0.2%).

## 3. Methods

### 3.1. Efavirenz Pharmacokinetics

Efavirenz (EFV) is a non-nucleoside reverse transcriptase inhibitor that is frequently used in first-line therapy in resource-constrained regions in combination with emtricitabine (FTC) and tenofovir disoproxil fumerate (TDF) for treatment of HIV infection. EFV is a small (molecular mass: 315.6 g/mol) lipophilic (LogP ≈ 4) compound that is highly bound to plasma proteins (human serum albumin and α-1-acid glycoprotein). The unbound fraction of the drug in human plasma (*f*_*u*_) is <1% (Almond et al., 2005; Fayet et al., 2008; Burhenne et al., 2010; Avery et al., 2011, 2013a). Efavirenz is a known inducer of various CYP-P450 enzymes (Fichtenbaum and Gerber, 2002), including *CYP2B6*, which is the main enzyme mediating its own metabolism (Ward et al., 2003; Ogburn et al., 2010). Moreover, it is known that CYP-P450 polymorphisms, in particular *CYP2B6* can lead to large inter-individual variations in EFV concentrations (Orrell et al., 2016). We derived statistical models for the inter-individual variability in plasma pharmacokinetic profiles, particularly taking CYP P450 polymorphisms (*CYP2B6* and *CYP2A6*) in a representative population (ENCORE 1) into account. Furthermore, we modeled metabolic autoinduction and established the relationship between plasma- and target-site concentrations.

#### 3.1.1. Pharmacokinetic Model Building

The population pharmacokinetic analysis of ENCORE 1 has previously been reported (Dickinson et al., 2015, 2016). Briefly, nonlinear mixed effects modeling using NONMEM (v. 7.2; ICON Development Solutions, Ellicott City, MD, USA) was applied to the efavirenz concentration-time data using FOCE-I. The impact of the following covariates on efavirenz apparent oral clearance (CL/*F*_bio_) was investigated: age, weight, fat-free mass (FFM), body mass index (BMI), sex, ethnicity and CYP P450 genotypes *CYP2B6* 516G>T, *CYP2B6* 983T>C, *CYP2B6* 15582C>T, CYP2A6^*^9B, CYP2A6^*^17, CYP3A4^*^22, *NR1I3* 540C>T and *NR1I3* 1089T>C. Specifically, of the 606 patients with PK data, 95% had a blood sample for genotyping (n=574), although amplification failed for a small number of individuals (*CYP2B6* 15582C>T and CYP3A4^*^22, n=1; CYP2A6^*^17, n=2; CYP2A6^*^9B, n=4). To drive the PrEP simulations, the final model was used to simulate PK parameters of 1000 virtual patients receiving efavirenz using the same distribution of significant covariates as the original dataset. PK parameters of all virtual patients are summarized in Supplementary Table 1.

Efavirenz concentrations over time were best described by a 1 compartment model parameterized by apparent oral clearance [population value of CL/*F*_bio_; estimate (RSE%): 11.9L/h (2.4%) for the reference (wild-type) CYP genotype for all four SNPs; *CYP2B6*: 516G>T/983T>C/CYP2A6^*^9B/^*^17 of a 70kg weighing individual], apparent volume of distribution [population mean *V*/*F*_bio_; 282 L (5.2%)] and absorption rate constant *k*_*a*_ fixed to a value of 0.6h^−1^ (Arab-Alameddine et al., 2009):

(1) $$\begin{array}{r} \frac{d}{dt}Z_{1}=-k_{a}\cdot Z_{1} \end{array}$$

(2) $$\begin{array}{r} \frac{d}{dt}D_{i,j}=\frac{k_{a}\cdot Z_{1}}{V_{i}/F_{\text{bio}}}-\frac{\text{C}{\text{L}}_{i,j}\left(t\right)/F_{\text{bio}}}{V_{i}/F_{\text{bio}}}\cdot D_{i,j} \end{array}$$

whereby *Z*_1_ denotes the amount of drug in the dosing compartment. The variable of interest is the concentration in the blood plasma (central compartment), i.e., *D*. Dosing events were modeled as impulse inputs, with

(3) $$\begin{array}{r} Z_{1,t}=Z_{1,t}+{\text{dose}}_{k}, \end{array}$$

whenever the current simulation time *t* coincided with a dosing event τ_*k*_. In the equations above, CL_*i, j*_(*t*)/*F*_bio_ denotes the bioavailability-adjusted, individual drug clearance at occasion *j* and *k*_*a*_ denotes the rate of drug uptake. The term *V*_*i*_/*F*_bio_ is the bioavailability-adjusted volume of distribution of individual *i*. Interindividual and interoccasion variability was supported on CL/*F*_bio_ [36.6% (10.8%) and 21.0 (27.7%), respectively] and residual error was defined by a proportional model [20% (8.6%)]. CL/*F*_bio_ and *V*/*F*_bio_ were allometrically scaled by weight (centered on 70 kg) and *CYP2B6* 516G>T/983T>C/CYP2A6^*^9B/^*^17 composite genotype significantly reduced efavirenz CL/*F*_bio_ between 4.5-82%, depending on allele combinations, compared to the reference genotype. Pharmacokinetic parameters for a 70 kg individual with reference genotype are summarized in Table 1. Overall, there were 16 genotype subgroups (Supplementary Table 2). Grouping of patients as extensive, intermediate or slow metabolisers (see below) as part of the modelling process (or after the final model was obtained) did not impact individual parameter estimates. The reduced genotype groups were defined as follows: (i) extensive metabolisers, (ii) intermediate metabolisers and (iii) slow metabolisers as detailed in Dickinson et al. (2015).

**Table 1:** Pharmacokinetic parameters.

**Parameter**	**Value**	**Unit**	**Parameter**	**Value**	**Unit**
CL_*ss*_/*F*_bio_	11.9	L/h	*V*/*F*_bio_	282	L
α	0.58	–	σ	0.20	–
*CV*_IIV_(CL_*ss*_/*F*_bio_)	36.6	%	*CV*_IOV_(CL_*ss*_/*F*_bio_)	21.0	%

The table displays the pharmacokinetic parameter estimates for a 70 kg individual with reference genotype (reference: CYP2B6, pos. 516:GG, pos. 516:TT and for CYP2A ^*^9B and ^*^17: CC/CC or CC/CT or CC/TT or CA/CC or CA/CT or AA/CC or AA/CT Dickinson et al., 2015). Inter-individual variability (IIV), as well as inter-occasional variability (IOV) was included on drug clearance CL/F_bio_. These parameters were log-normal distributed with coefficient of variation [%] $CV=100\cdot \sqrt{e^{\sigma^{2}}-1}$, where σ^2^ is the variance of the associated normal distribution. Weight was considered to affect $\text{C}{\text{L}}_{ss}\left(i\right)/F_{\text{bio}}=\text{C}{\text{L}}_{ss}/F_{\text{bio}}\cdot {\left(\text{weight}\left(i\right)/70\right)}^{0.75}$ and the volume of distribution V(i)/F_bio_ = V/F_bio_·(weight(i)/70) through allometric scaling. Residual variability was described by a proportional error model (σ = 0.2).

For initial model building clearance was assumed to reflect values *after* metabolic autoinduction since pharmacokinetic data was collected at weeks 4 and 12 of therapy. In the following, we consider the autoinduction explicitly, since it affects PrEP efficacy shortly after its initiation (e.g., “PrEP on demand”).

#### 3.1.2. Metabolic Autoinduction

In our work, we modeled metabolic autoinduction similarly to the model proposed by Zhu et al. (2009). We defined the term α as the ratio of the mean clearance on day 1 to the mean clearance at steady state (after autoinduction). The clearance ratio α is then computed as $\alpha =\frac{{𝔼}_{i}\left(\text{C}{\text{L}}_{i,t_{0}}\right)}{{𝔼}_{i}\left(\text{C}{\text{L}}_{i,SS}\right)}$ where the *average* clearance on the first day 𝔼_*i*_(CL_*i*,_*t*__0__) = 5.76L/h was taken from Zhu et al. (2009) and the *average* clearance at steady state 𝔼(CL_*i,SS*_) = 9.86L/h was computed from the virtual patient population (Supplementary Table 1), deriving α = 0.58. For each virtual patient generated from the population pharmacokinetic model, the individual clearance at steady state was available and the clearance at day 1 was computed using CL_*i*,_*t*__0__ = α·CL_*i, SS*_. Zhu et al. (2009) proposed a model for time-dependent autoinduction that we used herein

(4) $$\begin{array}{r} CL_{i}\left(t\right)=\text{C}{\text{L}}_{i,t_{0}}+\left(\text{C}{\text{L}}_{i,SS}-\text{C}{\text{L}}_{i,t_{0}}\right)\cdot \frac{t-t_{0}}{\left(t-t_{0}\right)+T_{50}} \end{array}$$

where CL_*i*_(*t*) is the individual clearance rate at the time *t* and *t*_0_ is the time of the first EFV dose. CL_*i*_(0) and CL_*i, SS*_ represent the clearance rates at day 1 and at steady state. The term *T*_50_ = 245h (Zhu et al., 2009) is the time where the clearance rate reaches half of its steady-state value.

#### 3.1.3. Target-site Concentrations

The general perception is that only the free/unbound intracellular concentration at the site of action (intracellular space) is available to exert an antiviral effect (Smith et al., 2010). For highly lipophilic drugs like EFV, passive diffusion may be the dominating transport mechanisms and therefore the *unbound/free* drug concentrations are identical on both sides of biomembranes, whereas the relation between the *total* concentrations can be computed by considering unspecific drug retention by e.g. binding to plasma proteins or lipids. These assumptions are implemented in so called partition coefficient models commonly used in physiologically based pharmacokinetic modeling, see von Kleist and Huisinga (2007) for an overview. To test whether EFV is dominantly transported into cells by passive diffusion/equilibrating transport we implemented partition coefficient models and compared the predictions with intracellular concentration measurements in Supplementary Text 1. We found overwhelming evidence for passive diffusion/equilibrating transport as the dominating mechanism of cellular drug uptake. Moreover, under passive diffusion and unspecific drug retention, there is a direct proportionality between plasma concentrations and concentrations at the site of action. This proportionality implies that we can model the effect of EFV based on plasma drug concentrations (derivations in Supplementary Text 1).

### 3.2. Direct Effects

We modeled the *direct* effect of efavirenz using the sigmoidal Emax-equation (Chou, 2006)

(5) $$\begin{array}{r} \eta_{D}\left(t\right)=\frac{D_{t}^{m}}{\text{I}{\text{C}}_{50}^{m}+D_{t}^{m}}, \end{array}$$

where *D*_*t*_ is the plasma concentration of the drug at time *t*, which is directly proportional to the target-site concentration (previous section and Supplementary Text 1) and the term IC_50_ and *m* denote the plasma concentration at which the targeted process is inhibited by 50% and a hill coefficient (Shen et al., 2008), respectively. Parameters are displayed in Table 2 for wild type, K103N, Y181C and G190S mutants together with their standard deviation. Note that the equation above couples the stochastic viral dynamics (below) to the deterministic pharmacokinetics (above). The hill coefficient *m* and 50% inhibitory concentration IC_50_ have been measured *ex vivo* using single-round infection assays in primary human peripheral blood mononuclear cells, supplemented with 50% human serum for *wild-type* HIV and various resistance mutations (K103N, Y181C and G190S) (Shen et al., 2008; Sampah et al., 2011). Since the *ex vivo* assay was performed with 50% human serum, the measured IC_50_ has to be corrected for protein content, since the drugs' potency might otherwise be overestimated, particularly for highly protein bound drugs like EFV. The IC_50_ correction is demonstrated in Supplementary Text 1, together with a sensitivity analysis with regard to uncertainties in measuring the unbound fraction of EFV in human blood plasma.

**Table 2:** Pharmacodynamic parameters.

**Strain**	**IC_50_ (± sd)**	**m (± sd)**	**f**
*wild type*	5.4 (± 0.9)	1.69 (±0.08)	1
Y181C	2.8·IC_50_(wt)	0.9·*m*(wt)	0.78
K103N	89.1·IC_50_(wt)	0.83·*m*(wt)	0.74
G190S	72.1·IC_50_(wt)	0.6·*m*(wt)	0.24

*The table displays the pharmacodynamic parameters for wild type (Shen et al., 2008) and different viral mutants (Sampah et al., 2011). The hill coefficient m (unit less) was assumed to be normal distributed and IC_50_ values (nmol/L) were assumed to be log-normal distributed (Jilek et al., 2012). Parameters were corrected for protein binding as outlined in Supplementary Text 1. Parameter f (unit less) denotes the fitness of the respective strains. The respective parameter distributions for the mutants (IC_50_, m) were computed by assuming an identical coefficient of variation as compared to the wild type*.

### 3.3. Viral dynamics.

We adopted the viral dynamics model described in von Kleist et al. (2010) and von Kleist et al. (2011). Long-lived and latently infected cells are only implicitly considered (outlined at the end of the section), motivated by the observation that transmitted viruses are not macrophage-tropic (Isaacman-Beck et al., 2009; Ping et al., 2013) and in line with related modeling approaches (Tan and Wu, 1998; Stafford et al., 2000; Perelson, 2002; Tuckwell et al., 2008; Conway et al., 2013). Although this model is a simplified representation of the molecular events happening during virus replication, it allows to accurately and mechanistically describe the effect of all existing antiretroviral drug classes on viral replication, as previously reported in (e.g., Duwal and von Kleist, 2016), and can be parameterized by *in vitro* and *clinical* data, Table 3. The modeled viral replication cycle consists of free infectious viruses *V*, uninfected T-cells (T_u_), early infected T-cells (T_1_) and productively infected T-cells (T_2_). Early infected T-cells (T_1_) and productively infected T-cells (T_2_) denote T-cells prior- and after proviral integration, respectively, where the latter produces virus progeny. During the onset of infection the number of viruses is relatively low and the number of uninfected T-cells T_u_ is fairly unaffected by viral dynamics (Perelson et al., 1993; Tan and Wu, 1998; Pearson et al., 2011). We thus consider T_u_ = λ_T_/δ_T_ to be constant over the course of simulations. The stochastic dynamics of viral replication after virus exposure are then defined by six reactions:

(6) $$\begin{array}{rcl} a_{1}\left(D_{t}\right)= & \left(\text{C}{\text{L}}_{\text{V}}+\text{C}{\text{L}}_{\text{T}}\left(D_{t},\text{mut}\right)\cdot {\text{T}}_{\text{u}}\right)\cdot V_{t} & \text{(clearance of free virus;}V\to *\text{)} \end{array}$$

(7) $$\begin{array}{rcl} a_{2}= & \left(\delta_{\text{PIC}}+\delta_{{\text{T}}_{1}}\right)\cdot {{\text{T}}_{1}}_{,t} & \text{(clearance of early infected cell;}{\text{T}}_{1}\to *\text{)} \end{array}$$

(8) $$\begin{array}{rcl} a_{3}= & \delta_{{\text{T}}_{2}}\cdot {{\text{T}}_{2}}_{,t} & \text{(clearance of late infected cell;}{\text{T}}_{2}\to *\text{)} \end{array}$$

(9) $$\begin{array}{rcl} a_{4}\left(D_{t}\right)= & \left(1-\eta_{D}\left(t\right)\right)\cdot \beta \cdot f\left(\text{mut}\right)\cdot {\text{T}}_{\text{u}}\cdot V_{t} & \text{(infection of a susceptible cell;}V\to {\text{T}}_{1}\text{)} \end{array}$$

(10) $$\begin{array}{rcl} a_{5}= & k\cdot {{\text{T}}_{1}}_{,t} & \text{(proviral integration;}{\text{T}}_{1}\to {\text{T}}_{2}\text{)} \end{array}$$

(11) $$\begin{array}{rcl} a_{6}= & N_{\text{T}}\cdot {{\text{T}}_{2}}_{,t} & \text{(production of virus;}{\text{T}}_{2}\to \text{V}+{\text{T}}_{2}\text{)}, \end{array}$$

with $\text{C}{\text{L}}_{\text{T}}\left(D_{t},\text{mut}\right)=\left(\frac{1}{\rho_{\text{rev}}}-\left(1-\eta_{D}\left(t\right)\right)\right)\cdot \beta \cdot f\left(\text{mut}\right)$ in Equation (6), as outlined in von Kleist et al. (2010) where ρ_*rev*_ = 0.5 denotes the probability to successfully complete reverse transcription in the absence of inhibitors (Pierson et al., 2002; Zhou et al., 2005) and *f*(mut) denotes the fitness of the mutant. Free viruses can be cleared within T-cells during unsuccessful infection with rate CL_T_ by destruction of essential viral components of the reverse transcription-, or pre-integration complex (Pierson et al., 2002; Zhou et al., 2005) or it may get cleared by the immune system with a rate constant CL_V_. Further, the term β represents the lumped rate of infection of T-cells, including the processes of virus attachment to the cell, fusion and reverse transcription, leading to an early infected cell T_1_, before proviral integration. The term *k* denotes the rate by which early infected T_1_ cells are transformed into productively infected T_2_ cells, involving proviral integration and cellular reprogramming. The term N_T_ denotes the rate of production of infectious virus progeny by productively infected T_2_ cells. The terms δ_T_1__ < δ_T_2__ denote the rates of clearance of T_1_ and T_2_ cells, respectively, and δ_PIC_ denotes the rate of intracellular destruction of the pre-integration complex. Parameters for the viral model are summarized in Table 3. In this article, we study distinct prophylactic schemes with the non-nucleoside reverse transcriptase inhibitor efavirenz. Reverse transcriptase inhibitors act intracellularly on reverse transcription. In our viral dynamics model this translates into an increase of propensity function *a*_1_ and a proportional decrease in propensity function *a*_4_. Derivations and motivation of this mechanisms of action from first principles are given in von Kleist et al. (2010) (Supplementary Methods therein).

**Table 3:** Parameters used for the viral dynamics model.

**Parameter**	**Value**	**References**	**Parameter**	**Value**	**References**
λ_T_	2·10^9^	Wei et al., 1995	*k*	0.35	Zhou et al., 2005
δ_T_, δ_T_1__	0.02	Sedaghat et al., 2009	β	8·10^−12^	Sedaghat et al., 2008
δ_T_2__	1	Markowitz et al., 2003	N_T_	670	Sedaghat et al., 2009; von Kleist et al., 2010
δ_PIC_	0.35	Zhou et al., 2005; Koelsch et al., 2008	CL_V_	2.3	Tan and Wu, 1998; Tuckwell et al., 2008

*Excerpt from von Kleist et al. (2010), except for CL_V_, which assumed that virus clearance is smaller in virus-naive individuals compared to infected individuals, in line with Frank et al., 2011; Duwal et al., 2012. All parameters refer to the absence of drug treatment ∅. All parameters in units (1/day), except for λ (cells/day) and β (1/(day · virus))*.

### 3.4. Virus Exposure

Initial viral exposure after sexual intercourse occurs at tissue sites typically not receptive for establishing and shedding HIV infection (e.g., mucosal tissues). Hence, the virus needs to pass several physiological barriers to reach a replication enabling (target-cell) environment where infection can be established and from where it can shed systemically (Joseph et al., 2015). To determine realistic inoculum sizes after sexual exposure to HIV (initial states for hybrid stochastic simulations), we previously developed a data-driven statistical model linking plasma viremia in a transmitter (VL) to the initial viral population *Y*_0_ in a replication-enabling environment (Duwal et al., 2016) (Supplementary Note 4 therein for details) precisely capturing average per contact transmission rates for various types of exposure. In brief, we assume a binomial model

(12) $$\begin{array}{r} P\left(Y_{0}=V|\text{VL}=\nu \right)=\left(\begin{array}{c} \left[\nu^{m}\right]\\ n \end{array}\right)\cdot r^{n}\cdot {\left(1-r\right)}^{\left[\nu^{m}\right]-n} \end{array}$$

where [·] is the nearest integer function, *m* = log_10_(2.45) is given by Wilson et al. (2008) and the *success probability r* was estimated in a previous work (Duwal et al., 2016) (Supplementary Note 4 therein), e.g., $r_{\text{homo}}=3.71\cdot 10^{-3}$ for homosexual- and $r_{\text{hetero}}=3.63\cdot 10^{-4}$ for heterosexual exposure. The parameter VL denotes the viral load in a potential transmitter (assumed to be log-normal distributed with μ = 4.51, σ = 0.98 (Duwal et al., 2016)). In this model, the *success probability r* summarizes both the extent of local exposure, as well as the probability of passing all bottlenecking physiological barriers and reaching a replication enabling target cell compartment. Herein, we used the “exposure model” to compute drug efficacy estimates after sexual exposure presented in **Figures 3**, **4**.

### 3.5. Numerical Simulation

We use the exact numerical simulation scheme proposed in Duwal et al. (2018). Briefly, the modeled system is split into stochastic reactions describing viral dynamics and a set of ordinary differential equations describing individual EFV pharmacokinetics after drug administration, including covariates (e.g., CYP2B6 polymorphisms), autoinduction and the relationship between plasma- and target-site concentrations outlined above. In our approach EFV pharmacokinetics affect certain stochastic reaction propensities as outlined in Equations (6), (9). This hybrid system is then simulated using the numerically exact EXTRANDE algorithm (Voliotis et al., 2016) and hybrid trajectories are classified as extinction events when all viral compartments are cleared. On the other hand, trajectories were considered infections if (i) either long-lived- or latently infected cells emerged, or if (ii) the trajectories left an *extinction simplex* (ϵ = 0.0001), meaning that it becomes unlikely (probability ≤ ϵ) that the virus will eventually be cleared (details provided in Duwal et al., 2018).

### 3.6. Prophylactic Efficacy of a Drug Regimen

Our goal is to estimate the prophylactic efficacy φ of a particular medication regimen *S*_*D*_. The prophylactic efficacy denotes the reduction in infection risk *per contact*, with φ = 100% indicating complete protection and φ = 0% indicating no change in infection risk.

(13) $$\begin{array}{r} \varphi \left(Y_{0},S_{D}\right)=1-\frac{P_{\text{I}}\left(Y_{0}|S_{D}\right)}{P_{\text{I}}\left(Y_{0}|\emptyset \right)}\left(\text{prophylactic efficacy}\right), \end{array}$$

where *P*_I_(*Y*_0_|*S*_*D*_) and *P*_I_(*Y*_0_|∅) denote the virus infection probabilities for a particular prophylactic scheme *S*_*D*_ and in the absence of prophylactic drugs (∅), respectively, for initial state $Y_{0}={\left[V,{\text{T}}_{1},{\text{T}}_{2}\right]}^{T}$ (number of viral particles, early- and late infected cells in a replication-enabling compartment). The probabilities *P*_I_(*Y*_0_|*S*_*D*_) are approximated by the number of simulations that were classified as *infection events* divided by the *total* number of hybrid stochastic simulation runs for each particular prophylaxis scheme *S*_*D*_ during PrEP, PrEP “on demand” and PEP simulations. *P*_I_(*Y*_0_|∅) can be computed using the analytical formulas derived in Duwal et al. (2019).

###  Simulation of Pre- and Post-Exposure Prophylaxis

Codes were written in MATLAB R2018b (MathWorks, Natick, MA; v. 9.5, including the statistics toolbox). Individual pharmacokinetic model parameters were drawn from the distributions defined by the parameter estimates from the final efavirenz population pharmacokinetic model (Table 2), generating 1000 virtual patients (Supplementary Table 1). We then simulated individual pharmacokinetic profiles for the prophylactic schedule *S*_*D*_ under consideration using ode113 in MATLAB. To simulate different adherence levels, a sequence of uniformly distributed random numbers with $r_{i}\sim U\left(0,1\right)$ was drawn and the *i*th dose was missed if *r*_*i*_ > adherence level.

The number of viruses to be inoculated was drawn from the virus exposure model, where we first sampled the viral load in a potential transmitter ($\log_{10}\text{VL}\sim N\left(4.51,0.98\right)$) and then used the virus load in the transmitter to determine the number of viruses *V*_0_ entering a replication-competent compartment in the virus-exposed individual using Equation. (12). Samples with *V*_0_ = 0 were rejected (they do not contribute to the infection risk). For once-daily PrEP simulations with different adherence levels, a time of virus exposure was randomly drawn within a 3 month interval starting at day 31 after PrEP initiation. The corresponding drug concentrations at this time and the number of transmitted viruses reaching a target cell compartment were used as the initial states for EXTRANDE and simulated until stopping criteria were satisfied (either virus clearance or infection). For “PrEP on demand” simulations, the time of virus exposure was fixed as indicated in the corresponding graphics. In the case of PEP, virus was inoculated as stated above and the stochastic viral dynamics were simulated in the absence of drugs until the time of PEP initiation (to determine the initial condition of the system), and henceforth simulated until a stopping criterium was reached.

In total, for each prophylactic scenario, 10000 simulations were run and *P*_I_(*Y*_0_|*S*_*D*_) was computed as the fraction of simulations that resulted in infection.

## 4. Results

### 4.1. Pharmacokinetics

The standard EFV dose used in treatment is 600 mg once daily taken orally. However, this dose is associated with neurotoxic effects (Rakhmanina and van den Anker, 2010; Apostolova et al., 2015), which could be prohibitive when using EFV as prophylaxis. Notably, neurotoxicity is associated with EFV plasma concentrations (and CYP2B6 polymorphism) (Rakhmanina and van den Anker, 2010). Therefore, a reduced, 400 mg dose has recently been explored, significantly reducing the risk of neurotoxicity while maintaining sufficient antiviral effects (ENCORE1 Study Group, 2014; ENCORE1 Study Group et al., 2015).

**Figure 1:**
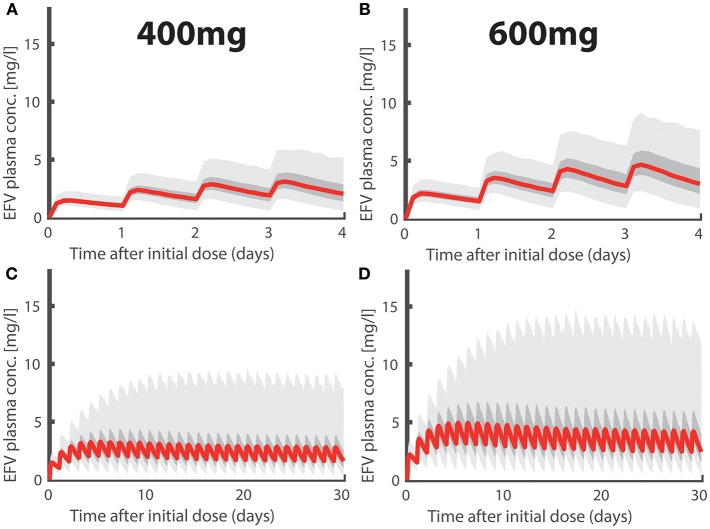
Efavirenz Pharmacokinetics. Population plasma pharmacokinetics for the first four days after intake of **(A)** 400- and **(B)** 600-mg daily oral EFV. Long-term pharmacokinetics of **(C)** 400- and **(D)** 600-mg daily oral EFV due to metabolic autoinduction. Light gray regions encompass 95% of individual PK predictions, whereas dark gray areas encompass 50% of the predictions (quartiles). The thick red line marks the median pharmacokinetic profiles.

In Figures 1A,B we depict simulated pharmacokinetics of once daily oral EFV with 400 and 600 mg. EFV was quickly absorbed with a median *t*_max_ ≈ 5.90h (95% CI: 4.56–7.88) and has a long median half life *t*_1/2_ ≈ 35.57h (CI: 14.28–125.26) at day 1 and a median half life *t*_1/2_ ≈ 20.77h (CI: 8.34–73.15) after metabolic autoinduction, in agreement with the literature (Avery et al., 2011, 2013a; Dickinson et al., 2015). Due to its linear pharmacokinetics, the dose reduction 600 → 400 mg resulted in a concentration reduction of ≈2/3 for the 400mg dosing regime. In Figures 1C,D, we show the long-term pharmacokinetics after multiple dosing. Two things come to mind: (i) after an initial plateau phase (4–5 doses), concentrations tend to decrease, due to metabolic autoinduction, reaching median trough levels of ≈ 1.35 and ≈ 2.02 mg/L (95% CI: 0.30–6.55 and 0.45–9.83mg/L) in the 400- and 600mg dosing regime, respectively. (ii) The variability in the predicted pharmacokinetic profiles increases after multiple dosing with some individuals achieving concentrations >10 (mg/L) (light grey area indicating the 95% range). This observation is attributable to genetic polymorphisms affecting some individuals of our virtual patient cohort that slowly metabolize EFV. Interestingly, there is clinical evidence that some individuals, particularly slow metabolisers, achieve concentrations >10 (mg/L), and that the proportion of these individuals is much higher for the 600 mg regimen (Dickinson et al., 2015). In our simulations, 11.3% in the 600 mg group eventually exceed concentration of 10 mg/L, whereas it is only 2.5% in the 400 mg group. If EFV toxicity is proportional to exposure, as suggested by Rakhmanina and van den Anker (2010), this may indicate that dose reduction could significantly reduce the risk of adverse effects. But is it also protective against infection?

### 4.2. Concentration-prophylaxis Profile

**Figure 2:**
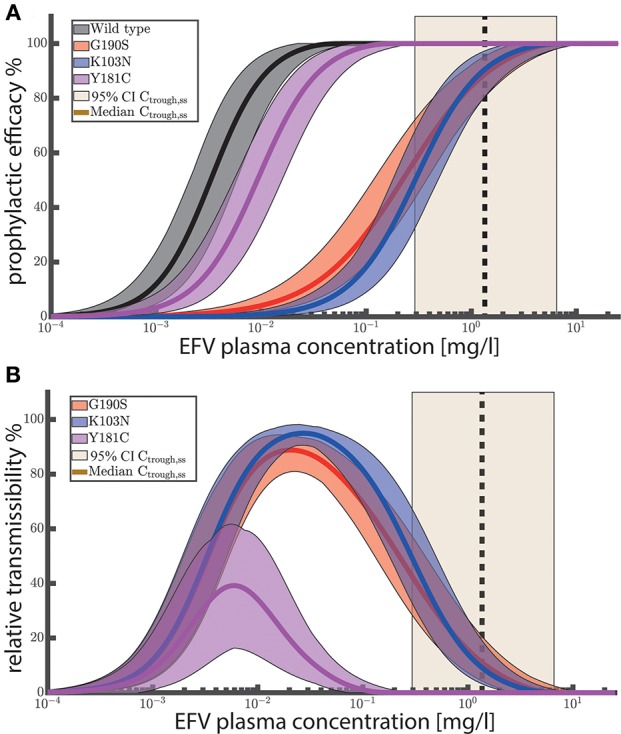
**(A)** Prophylactic efficacy against different viral genotypes. Predicted efficacy ranges are depicted as colored shaded areas, with superimposed mean efficacy estimates as solid lines. Ninety-five percent confidence intervals of steady-state EFV trough (pre-dose) concentrations *C*_trough,ss_ for 400 mg EFV after once daily dosing are depicted as light yellow areas with the vertical black dashed line marking the median trough concentration. **(B)** Relative transmissibility φ_wt_−φ_mut_ of mutant viruses. Colored areas depict the ranges of relative transmissibility and solid lines indicate the mean relative transmissibility. Yellow shaded areas depict the 95% CI of EFV trough (pre-dose) concentrations after 400 mg once daily at steady state. Black dashed vertical lines indicate the corresponding median trough concentrations. The prophylactic efficacy against a single virus was computed using the analytical solutions provided in Duwal et al. (2018) (Equation 19–21 therein) for 1000 logarithmically spaced concentrations between 10^−4^ and 25 mg/L using 1000 sampled values for IC_50_ and *m* per viral genotype (Table 2). For each viral genotype, IC_50__assay_ and *m* were sampled from a log-normal and normal distribution respectively as stated in the caption of Table 2. IC_50__assay_ were corrected for plasma binding using *f*_u,plasma_ = 0.2% to obtain IC_50__plasma_, as outlined in Supplementary Text 1. Changes in drug sensitivity for the mutants considered the fold changes stated in Table 2. Fitness defects of the mutants were considered using β(mut) = *f*(mut)·β(wt).

We used the analytical solutions presented in Duwal et al. (2019) to compute concentration-prophylaxis profiles φ(*Y*_0_, *S*_*D*_) assuming a single virus particle enters a replication-enabling compartment ($Y_{0}={\left[1,0,0\right]}^{T}$), see Figure 2A. The reason is that the virus exposure model (Methods section) predicts that in most cases only a single virus enters a replication-competent compartment after (homo-/hetero-)sexual exposure, if a virus manages at all to pass the various bottlenecking physiological barriers after sexual exposure. Besides the *wild-type* virus, we also show the prophylaxis profile against transmitted drug resistance with viruses carrying EFV resistance mutations G190S, K103N and Y181C (Rhee et al., 2003). As a visual guide, the shaded areas mark the 95% trough (pre-dose) concentration ranges achieved at plateau and after metabolic autoinduction for once daily 400 mg efavirenz (computed using the POP-PK model).

Figure 2A suggests that once daily EFV (with 400 mg) provides complete protection against HIV infection after exposure to *wild-type* virus and resistant viruses carrying the Y181C mutation. After exposure to the G190S and K103N mutants, >>50% protection is provided by once daily 400 mg EFV and >>60% protection by the 600 mg regime. Since selection of drug resistant variants is a major concern, we evaluated the relative transmissibility of mutant viruses when compared with *wild-type* virus as φ_wt_−φ_mut_ in Figure 2B. The figure can be interpreted as follows: At low concentrations there is no reduction in infection if an individual was exposed to *wild-type* and/or mutant virus. At an intermediate concentration range (between 0.001 and 0.1 for Y181C and between 0.001 and 1 mg/L for K103N, G190S, respectively), infections with the *wild type* would be prevented, while the prophylaxis cannot, or only partially reduce the infection risk after exposure to mutant virus. The maximum corresponds to the maximal difference in risk reduction, meaning that resistant virus is more likely transmitted than the *wild type*. At very high EFV concentrations, the infection risk with both *wild type* and mutant is reduced. Importantly, when inspecting (population) *median* EFV trough (pre-dose) concentrations after 400 mg once daily dosing (dashed vertical black line in Figure 2B) , we can see that the relative transmissibility of the Y181C mutant is zero, while the relative transmissibilities of the G190S and K103N mutants are less than 20%. The analysis suggests that the typical concentration ranges achieved after once daily EFV do not, or just slightly, favor resistance transmission over *wild type* for the considered single-substitution mutants. Note that these mutations decrease EFV susceptibility by ≈ 90 fold, Table 2. However, clinically derived isolates may contain multiple substitutions and confer even higher levels of EFV resistance.

Since poor drug adherence may give rise to lower EFV concentrations and since it is a major factor confounding the clinical efficacy of Truvada (Haberer et al., 2015), we next set out to test whether similar issues are to be expected for 400 mg oral EFV for pre-exposure prophylaxis, or when EFV is used “on demand” and post-exposure.

### 4.3. Once-daily PrEP With 400 mg EFV

**Figure 3:**
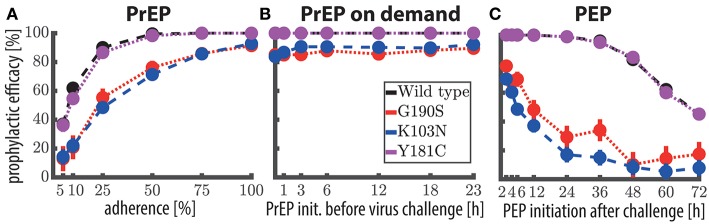
Prophylactic efficacy of EFV against the *wild-type* and resistant virus using different prophylaxis schemes. **(A)** Predicted prophylactic efficacies for once daily 400 mg oral EFV PrEP with different levels of adherence. For example in the 25% adherence scheme, each dosing event was randomly missed with 75% chance. Colored dots mark the median predicted prophylactic efficacy and error bars mark the 95% confidence interval (computed using Greenwood's formula), considering variabilities in pharmacokinetic, as well as pharmacodynamic parameters. **(B)** Predicted prophylactic efficacy of 400 mg oral EFV during “PrEP on demand” (3 doses) depending on the time of PrEP initiation with respect to viral encounter, respectively. **(C)** Predicted prophylactic efficacy of post-exposure prophylaxis (9 doses) with 400 mg oral EFV as a function of the time of PEP initiation after viral challenge. Simulations were conducted using the hybrid EXTRANDE method outlined in the Methods section. In total, 10,000 stochastic simulations were performed to estimate prophylactic efficacy for each condition (e.g., viral challenge with K103N during chronic PrEP with 5% adherence is one condition).

The predicted prophylactic efficacy of once daily PrEP with 400 mg is shown in Figure 3A as a function of adherence after exposure to either *wild-type* virus or after exposure to drug resistant mutants. As can be seen, if at least 75% of doses were taken, complete protection against the *wild-type* virus and against the Y181C mutant was achieved. Notably, for these viral genotypes protection was >95% if 50% of the pills were taken and ≈ 90% when ≈25% of the pills were taken. In contrast, after exposure to resistant viruses carrying the G190S or K103N mutation, protection was >82% when at least 75% of the once daily 400 mg EFV pills were taken, gradually dropping to ≈ 50% protection when every fourth pill was taken.

We next wanted to assess how quickly the prophylactic protection vanishes, when consecutive EFV doses were missed (illustratively depicted in Supplementary Figure 1). In order to do so, we simulated 400 mg EFV-based once daily PrEP with 100% adherence. Subsequently, we computed how long it will take for the concentrations to drop below the respective 50%, or 90% protective levels (EC_50_, EC_90_). We computed that a median of 7 (CI: 2–32) consecutive doses need to be missed in order to provide less than 50% protection against *wild-type* virus. Correspondingly, 5 (CI: 1–26) consecutive doses need to be missed to provide less than 90% protection against *wild-type* virus.

### 4.4. “PrEP on Demand” With 400 mg EFV

Next, we evaluated whether 400 mg EFV “on demand” would protect against HIV infection. We tested an “on demand” dosing scheme similar to the one recently tested for Truvada-based PrEP (Molina et al., 2015): The first EFV dose was taken within a time window of 1–23 h *prior* to virus exposure and followed by two more doses, 24- and 48- hours after the initial dose. Our predictions indicate that EFV-based “PrEP on demand” provides complete protection against *wild-type* virus and against the Y181C single mutant, when initiated 1–23 h prior to virus exposure. Protection against the single mutants G190S and K103N was still >81% for 400 mg “PrEP on demand.” This surprisingly superior prophylactic efficacy of EFV “on demand” can be attributed to its rapid uptake and slow elimination. Particularly the latter ensures that virus gets eliminated when EFV is taken as “PrEP on demand.” The comparatively higher efficacy of “PrEP on demand,” when compared to once daily PrEP with low adherence can be explained as follows: In the case of once-daily PrEP, several *consecutive* dose intakes may be missed, which allows the EFV concentrations to fall below their respective EC_50_, EC_90_. If virus exposure occurs during these time windows of low EFV concentrations, infection may occur (illustrated in Supplementary Figure 1). In contrast, if all “PrEP on demand” pills are taken, concentrations will be above the EC_90_ at the time of exposure, and, due to the long half life of EFV remain above this value, until the virus is eliminated (which typically would happen ≤1 week post exposure Konrad et al., 2017).

### 4.5. Post-Exposure Prophylaxis With 400 mg EFV

**Figure 4:**
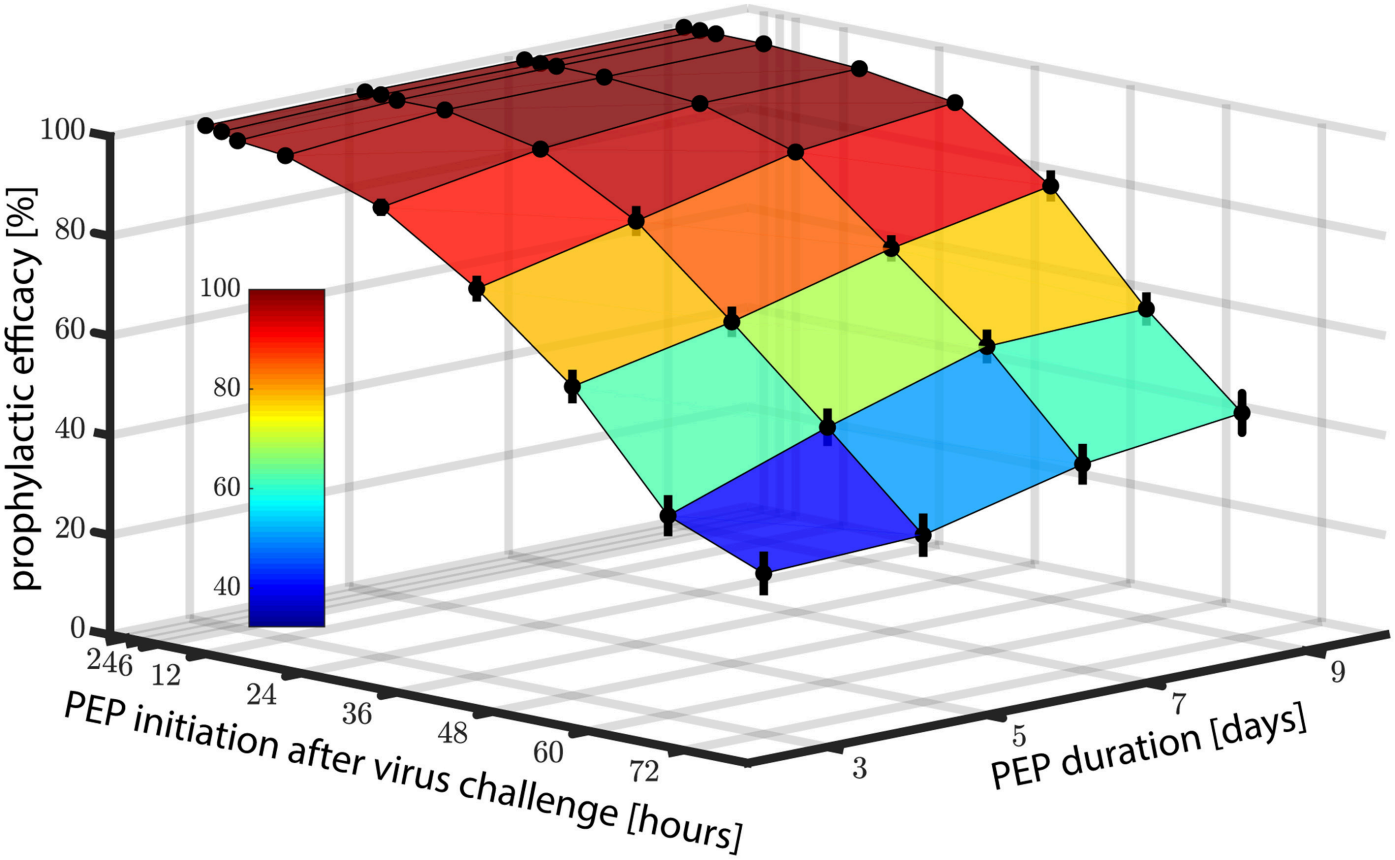
Prophylactic efficacy of *PEP* against *wild type* virus depending on both the time of PEP initiation and the number of subsequent 400 mg EFV doses taken. Error bars denote the 95% confidence intervals, computed using Greenwood's formula. Prophylactic efficacy was estimated for each condition based on 10,000 hybrid EXTRANDE simulations as outlined in the Methods section.

Motivated by the promising predictions regarding the use of EFV in pre-exposure prophylaxis, we also wanted to investigate whether EFV could prevent infection, if taken as post-exposure prophylaxis (PEP). In Figure 4, we show the predicted prophylactic efficacy of 400 mg oral EFV as a function of both the delay in PEP initiation and the duration of PEP after challenge with *wild-type* virus. In Figure 4 it becomes evident that it is more critical to initiate PEP early after exposure, than to prolong PEP duration. For example, when PEP is initiated as late as 72 h post virus exposure and the duration of PEP is three days (3 consecutive doses), the prophylactic efficacy was estimated to be ≈20%. If the duration of PEP was increased to 9 days, the prophylactic efficacy increases to only ≈40%. However, if PEP was initiated shortly after virus exposure (e.g., within 2 h), the prophylactic efficacy increases to 100%, even if the PEP duration was only 3 days.

As a midpoint, taking the first PEP dose within 24 h post-exposure resulted in prophylactic efficacies of >88, >94, >97 and >98% against the *wild type* for 3, 5, 7, and 9 dose intakes, respectively.

Next, we wanted to investigate in more detail the sensitivity of PEP efficacy towards the timing of PEP initiation in the *wild type* and drug resistant mutants. To simplify interpretation, we assumed a PEP duration of 9 days (9 doses). Predictions are shown in Figure 3C. PEP provided >98% protection against the *wild type* and the Y181C mutant when started within 12 h after virus exposure. Protection against viruses containing the G190S mutation was >21% using the same parameters, and >11% for the K103N mutant. These simulations indicate that EFV may potently protect against infection with *wild type* and the weakly resistant Y181C virus, when initiated within 24 h post expose. The prophylactic efficacy against transmitted, highly resistant viruses carrying the G190S or K103N mutation is insufficient for post-exposure prophylaxis.

## 5. Discussion

Truvada-based PrEP is being implemented in a number of countries (AVAC, 2019), however, there are two major limitations to its optimal use: (i) its costs (Keller and Smith, 2011), and (ii) its sensitivity to poor medication adherence (Haberer et al., 2015).

Current PrEP research focusses on overcoming adherence-related concerns, either in terms of promoting drug adherence, or through the development of novel long-acting drugs/drug formulations for HIV prophylaxis, that only require monthly drug administration (McGowan et al., 2016; Markowitz et al., 2017; McMillan et al., 2017). However, little has been done to investigate cost-efficient Truvada alternatives that may be affordable in low- and middle-income countries hit hardest by the epidemic.

A recent computational screen of the prophylactic potential of treatment-approved compounds for PrEP repurposing suggested that darunavir, efavirenz, nevirapine, etravirine and rilpivirine may more potently prevent HIV infection than Truvada at clinically relevant concentration ranges (Duwal et al., 2019). Of these candidates we set out to investigate efavirenz in more detail, since it is both inexpensive and readily available in most resource-constrained settings.

However, 600 mg EFV has been associated with neurotoxicity (Rakhmanina and van den Anker, 2010; Apostolova et al., 2015). Decloedt and Maartens (2013) and Siccardi et al. (2012) have previously suggested a connection between EFV metabolism and toxicity, indicating that slow metabolisers, who have higher plasma concentrations, also have a higher tendency to experience adverse effects (associations have been made between the major EFV metabolite and neurotoxicity). The direct association between plasma concentrations and CNS side effects has also been reported in Marzolini et al. (2001). Motivated by these studies, we explicitly considered genetic polymorphisms affecting EFV metabolism. Moreover, since EFV pharmacokinetics are linear, dose reductions would naturally lead to decreased EFV exposure (and consequently toxicity) as investigated in the ENCORE 1 trial (ENCORE1 Study Group, 2014; ENCORE1 Study Group et al., 2015), which suggested non-inferiority of the 400 mg EFV regimen with regard to treatment. Motivated by these results, we set out to investigate the prophylactic potential of 400 mg EFV.

Our simulations strongly suggest that 400 mg efavirenz can potently prevent infection with drug susceptible HIV, when used as once daily PrEP, during “PrEP on demand” and even as PEP, if initiated early enough after exposure (Figures 3, 4). Overall, these simulations suggest that EFV provides a good efficacy margin with respect to incomplete adherence and various event-driven dosing scenarios. Notably, if the association between EFV toxicity and metabolism is evident, it could also be envisioned that individuals that experience adverse effects may even further reduce EFV dosing. For example, using the POP-PK model, we predicted that the number of patients experiencing plasma concentrations of >10 mg/L following a 200mg once daily dosing regimen is only 0.1%.

However, our simulations also suggested that EFV-based post-exposure prophylaxis (PEP) may insufficiently protect against transmitted, highly resistant strains (K103N, G190S), as depicted in Figure 3C. We should also note that circulating resistant viruses may have multiple compensatory mutations that increase fitness and resistance through epistatic effects (Rath et al., 2013). Thus, their phenotypic attributes may deviate from laboratory strains with single point mutations that were evaluated in the present analysis and by Sampah et al. (2011). A recent study (Zazzi et al., 2018) highlighted high levels of NNRTI resistance particularly in South Africa, but it is unclear whether the analyzed NNRTI resistance mutations also confer high level resistance against EFV. Regarding high level resistance mutations, the Stanford database currently reports a prevalence π_untreated_ <<5% ^1^ in the *untreated* population in South Africa, mainly conferred by K103N. The prevalence of resistance mutations in *treated* individuals π_treated_ is much higher: K103N ≈30%, Y181C ≈20% and G190A/S ≈15% ^2^, but comparable to Truvada resistance mutations (M184V: 48–60%; K65R: 4–15%) in *treated* individuals. Notably, the overall risk of exposure to resistant strains would be much smaller than these numbers, as it is both determined by prevalence, as well as the probability of resistance-associated treatment failure in the donor at the moment of virus transmission, e.g., mathematically **Prob**.{exposure to res.} = **Prob**.{untreated.}·π_untreated_ + **Prob**.{treated.}· **Prob**.{failing due to resistance} ·π_treated_. The calculations state that resistance exposure from *treated* individuals may only originate from those *treated* individuals that fail on the treatment at the time of exposure, due to resistance emergence (if they are successfully treated at the time of exposure, they are non-contagious Cohen et al., 2011).

Another important aspect that is quantified in Supplementary Text 2 is resistance emergence in the exposed individual *prior* to PEP initiation. As can be seen in Supplementary Text 2, the probability of resistance emergence increases with the delay between virus exposure and PEP initiation. For example, we calculated that if PEP is initiated 3 days after exposure (72 h), the virus had either gone extinct or developed resistance with 38% probability. This *de novo* resistance may subsequently be selected by EFV, limiting future treatment options. On the other hand, if PEP is initiated within 12 hours, the probability of resistance emergence in the exposed individual *prior* to PEP is <0.01%. Thus, both in terms of lack of efficacy (Figure 4), as well as in terms of resistance (Supplementary Text 2), the window of opportunity with regards to PEP is short. Based on our simulations, PEP should be initiated as early as possible and is contraindicated if the suspected virus exposure occurred more than 3 days ago. The same considerations also apply for Truvada-based prophylaxis.

Our predictions regarding EFV prophylactic efficacy depend on (i) parameters of EFV potency (IC_50_) and (ii) the concentrations of EFV at the target site.

Regarding EFV potency, one limitation of our work is that we used parameters determined *ex vivo* (Shen et al., 2008; Sampah et al., 2011) using primary human peripheral blood mononuclear cells (PBMCs). These cell mixtures are commonly used as surrogate markers to determine drug efficacy, since they contain a large proportion of CD4^+^ T-cells (the primary HIV target cell type). Moreover, utilised parameters are generally in agreement with published values from other sources (Smith et al., 2001; Parkin et al., 2004; Avery et al., 2013b; Hu and Kuritzkes, 2014; Schauer et al., 2014) (after correction for protein binding; Supplementary Text 1).

Regarding the *relevant* target-site concentrations of EFV, there has been some debate since the *total* (protein bound and unbound) EFV concentrations in tissues have been reported to be highly heterogeneous (Thompson et al., 2015) and some studies have suggested associations between drug heterogeneity and incomplete HIV suppression (Fletcher et al., 2014), whereas others report high concentrations in tissues related to HIV exposure (Thompson et al., 2015). There are two main mechanisms that could explain heterogeneous drug distribution, which we discuss in detail:

a) Active transport (e.g. P-glycoprotein): In this case, the expression of transporters in particular cell types may cause concentration differences between distinct tissues. Notably, active transport would cause a difference in the *unbound* concentrations, which are available to exert an antiviral effect. As a consequence of active efflux, lower amounts of EFV may be available in some relevant target cells, giving rise to pharmacological sanctuaries relevant to EFV prophylaxis. A detailed analysis of EFV active transport (Burhenne et al., 2010) however revealed that it does not affect EFV intracellular concentrations. Moreover, EFV is a small (molecular mass: 315.6 g/mol) and highly lipophilic (LogP ≈ 4) compound that could rapidly cross biomembranes by passive diffusion. Thus, even if EFV was a substrate of cellular transporters, the *dominating* (i.e. fastest) mechanism mediating cellular uptake and efflux is probably passive diffusion. Furthermore, passive diffusion does not imply that the *total* (protein bound and -unbound) concentrations on either side of a biomembrane are equal, but rather implies that the *unbound* concentrations are equal. I.e. at each side of a biomembrane, EFV may be (un-)specifically retained by binding to biomolecules (lipids, proteins, see von Kleist and Huisinga, 2007 for an overview). However, since only the *unbound* concentration is available for drug-target interaction, EFV concentrations exerting antiviral effects would be identical in different cell types under passive diffusion.

b) Protein binding: EFV is highly (>99%) bound to plasma proteins (Boffito et al., 2003), mainly albumin and α−1-acid glycoprotein. Naturally, the concentrations of these proteins are magnitudes lower in tissues, which affects the amount of protein-bound EFV (and consequently the *total* concentrations). Studies that measure *unbound* drug concentrations lend strong support to this hypothesis. Avery et al. reported that the *unbound* EFV concentrations in plasma and semen (Avery et al., 2011) and in plasma and cerebrospinal fluid are nearly identical (Avery et al., 2013a). Importantly, considering albumin concentrations (calculations in Supplementary Text 1) in proposed sanctuary sites, we can precisely recover differences in *total* EFV concentrations reported, e.g., semen-to-plasma ratio: 3.4–5 % (Reddy et al., 2002; Avery et al., 2011) and cervical fluid-to-plasma ratio: 0.4% (Dumond et al., 2007). The fact that *unbound* plasma-, cerebrospinal fluid- cervical fluid and semen concentrations are nearly identical also suggests that EFV can cross the blood-brain, blood-testis and blood-uterine barrier.

In summary, these combined observations strongly argue that the distribution of EFV in tissues is governed by passive diffusion and (un-)specific binding to plasma proteins. In terms of PK-PD modeling, this implies that the *unbound* concentration in plasma are representative for the *unbound* concentration within target cells (CD4^+^ immune cells/T-cells; derivations in Supplementary Text 1). When *unbound* concentrations are proportionally related to the *total* concentrations, it also implies that EFV *total* plasma concentrations can be used as a marker of drug efficacy (Marzolini et al., 2001). As a cautionary note we want to add that there could still be additional unaccounted, specific barriers lowering EFV *unbound* concentrations in physiological sites relevant for establishing the initial infection upon sexual exposure to HIV-1 (male genital compartment, female genital compartment and rectum), which warrant further verification. However, based on the discussions above, we would strongly disagree with the statement by Dumond et al., that “agents such as efavirenz that achieve *total* genital tract exposures less than 10% of blood plasma are less attractive PrEP/PEP candidates” (Dumond et al., 2007). This simplistic criterium of selecting drug candidates ignores the drug's individual pharmacology, might only select drugs that are not extensively protein bound, or select highly protein-bound candidates merely as a function of genital albumin concentrations. Our simulations are however in line with a later study from the same group (Dumond et al., 2012), which find that the concentrations at the site of virus exposure (in Dumond et al., 2012 the female genital tract) are proportional to unbound plasma concentrations during *chronic dosing*. However, it is unclear after how many dosing events this equilibrium between plasma and target site concentrations is achieved. While plasma concentrations rapidly peak at a *t*_max_ of about 5.9 h, there could be a time-delay in building up concentrations at the site of infection, which could impair the efficacy of “PrEP on demand” and PEP (compare Figures 3B,C), in the sense that it becomes more important to initiate the respective protocols as early as possible. Notably, genital tract concentrations measured after the first dose in Dumond et al. (2007) are in line with our predictions, arguing for our modeling approach and for the presumed fast kinetics of cellular uptake by passive diffusion (see also Supplementary Text 1).

Another limitation of our study is that the parametrization of the PK model is based on data from HIV-infected individuals, while prophylaxis is intended for healthy individuals. In fact, it is unclear whether there are significant differences with respect to e.g., drug metabolism as a consequence of the infection status. For example, measured EFV plasma concentrations (400 mg once daily) in healthy individuals from Burhenne et al. (2010) are similar to those predicted herein. However, our model predicts large inter-individual variabilities due to pharmacogenomics (CYP 450 C2B6 polymorphisms). This hints toward the fact, that the pharmacokinetic differences between healthy vs. infected individuals could be small in comparison to the variability due to CYP polymorphisms. On the other hand, a study in healthy Ugandan individuals reports EFV concentrations (Mukonzo et al., 2009) that are considerably larger than predicted by our model. At the moment it is unclear whether differences are due to the infection status, or contributed to differences in ethnicity, weight, or co-medications: I.e. ethnicity (“black”) has been associated with lower EFV clearance (Barrett et al., 2002). However, it is unclear whether concentration differences are due to a higher proportion of poor metabolisers in Ugandans, as suggested by Mukonzo et al. (2009), or other factors. It is interesting to note here that “gender” was a significant co-variate in the Ugandan study whereas it was not associated with changes in EFV PK in the ENCORE 1 study (Dickinson et al., 2015). While the drug's half life is similar for ENCORE 1 patients (35.57h; CI: 14.28–125.26) and healthy men in the Ugandan study (Mukonzo et al., 2009) (37.3h in wild type and 54.7h in slow metabolisers), the drug's terminal half life in females in the Ugandan study (Mukonzo et al., 2009) was twice as large as that for men. For comparison, a meta-analysis of 16 phase I studies reports a difference of only 10% (Barrett et al., 2002), warranting further research to clarify the mechanistic sources of the discrepancy between the results from the phase I studies (Barrett et al., 2002), the Ugandan study (Mukonzo et al., 2009) and the data from ENCORE 1 (Dickinson et al., 2015).

Regarding possible co-medications, it is worth mentioning that efavirenz has a large drug interaction potential. For example, it has been shown in Fan et al. (2009) that certain herbal medicines might compete for CYP2B6 metabolisms raising plasma levels, potentially up to toxic ranges. In any case, toxicity in the context of EFV-PrEP remains to be elucidated clinically and it remains to be elucidated if even further dose reductions would be suitable for PrEP in particular populations. The present work provides a good starting point to support these decisions, e.g., based on the concentration-prophylaxis profiles presented in Figure 2.

Moreover, EFV is an inducer of many CYP enzymes (Fichtenbaum and Gerber, 2002), possibly altering the pharmacology of co-medications. Thus, co-medication with EFV-based PrEP might require careful monitoring. The Liverpool drug-interaction database provides an excellent overview over known effects of EFV on various co-medications (https://www.hiv-druginteractions.org/).

Overall, this mathematical modelling study argues for the experimental investigation of EFV as a cost-efficient alternative PrEP candidate based on its superior prophylactic efficacy and forgiveness to incomplete adherence and event-driven usage. However, further analysis emphasising on the safety of EFV in the context of PrEP/PEP is warranted.

## Data Availability

All data is contained in the manuscript.

## Author Contributions

SD and MvK conceptualized and designed the study. SD and DS performed the analysis on modeling prophylactic efficacy. LD performed the PK analysis. MvK wrote the manuscript with inputs from SD, DS, LD, and SK. All authors contributed to the analysis of the results.

### Conflict of Interest Statement

The authors declare that the research was conducted in the absence of any commercial or financial relationships that could be construed as a potential conflict of interest.

## Footnotes

^1^https://hivdb.stanford.edu/page/surveillance-map/

^2^https://hivdb.stanford.edu/cgi-bin/MutPrevBySubtypeRx.cgi

## Supplementary Material

Supplementary Figure 1 The figure shows an example of a concentration-time profile for chronic PrEP with 400 mg oral EFV and 25% adherence, where a temporal window for infection arises and EFV concentrations are insufficient for protection.

Supplementary Text 1 The supplementary text contains an in-depth analysis of EFV cellular uptake, providing support for the free drug hypothesis and for equilibrative transport or passive diffusion as the main cellular uptake mechanisms of EFV. Furthermore, it analyses whether the drug potency is sensitive to uncertainty in drug binding.

Supplementary Text 2 This supplementary text quantifies the probability of EFV resistance emergence prior to PEP initiation. We derive the probability of resistance emergence as a function of the time between virus exposure and start of post-exposure prophylaxis. The aim is to provide decision support on whether to start PEP after suspected exposure or not.

Supplementary Table 1 The table shows the individual pharmacokinetic parameters (CL_ss_/*F*_bio_, *V*/*F*_bio_ and *k*_*a*_) of all virtual patients.

Supplementary Table 2 The table shows the considered single nucleotide polymorphisms associated with EFV metabolism and how they were modeled to affect efavirenz apparent oral clearance in the population pharmacokinetic model.
