# Supplementary material for: The Utility of Efavirenz-based Prophylaxis Against HIV Infection. A Systems Pharmacological Analysis
Source: Front Pharmacol. 2019 Mar 13;10:199. doi: 10.3389/fphar.2019.00199 (PMC6424904; doi:10.3389/fphar.2019.00199)
Supplement: Supplementary Figure 1 — The figure shows an example of a concentration-time profile for chronic PrEP with 400 mg oral EFV and 25% adherence, where a temporal window for infection arises and EFV concentrations are insufficient for protection. [file Data_Sheet_3.PDF]

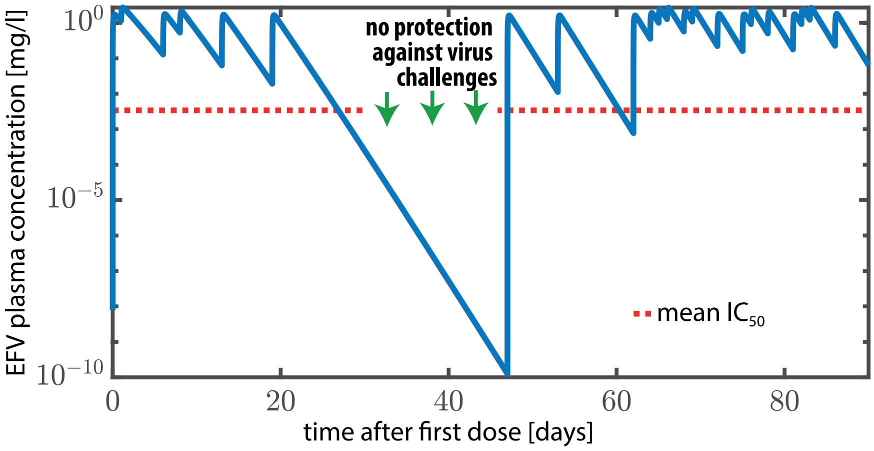

Figure 1: An example of a concentration-time profile after 400mg oral EFV with 25% adherence in virtual patient number 1 (CL_{ss}/F_{bio}= 8.04 mg/h; V_d/F_{bio}= 206.2 L; k_a=0.6 mg/h). The red dashed horizontal line depicts the mean IC_{50} value. As can be seen in this example, a temporal window may arise (roughly between 30 and 45 days post PrEP initiation), were EFV concentrations are insufficient for protection.
