# Supplementary material for: The Utility of Efavirenz-based Prophylaxis Against HIV Infection. A Systems Pharmacological Analysis
Source: Front Pharmacol. 2019 Mar 13;10:199. doi: 10.3389/fphar.2019.00199 (PMC6424904; doi:10.3389/fphar.2019.00199)
Supplement: Supplementary Text 1 — The supplementary text contains an in-depth analysis of EFV cellular uptake, providing support for the free drug hypothesis and for equilibrative transport or passive diffusion as the main cellular uptake mechanisms of EFV. Furthermore, it analyses whether the drug potency is sensitive to uncertainty in drug binding. [file Data_Sheet_1.PDF]

Supplementary Text S1.

S1.1 Cellular Uptake

We hypothesised that efavirenz might cross biomembranes by passive diffusion. In this case, an equilibrium between the *unbound* concentrations on each side of a biomembrane is rapidly established and the relation between the *total* concentrations on each side of the biomembrane is established by drug retention on each side of the biomembrane (e.g. specific or unspecific binding). This central idea is implemented in so called partition coefficient models, see [1] for an overview. To probe this hypothesis, we took data provided in [2] and compared it to predictions using the established partition coefficient model by Poulin and Theil [3]. Fig. S1.1 (left) shows the relation between *unbound* plasma- and cellular concentrations [2] along with the predictions using the Poulin-Theil model [3], which assumes passive diffusion of unbound drug and unspecific retention of drug in the intracellular space, whereas Fig. S1.1 (right) shows the relation between the *total* plasma- and cellular concentrations [4]. As can be seen, the data and predictions strongly argue for passive diffusion as the dominating cellular uptake mechanism. Moreover, in the case of passive diffusion and unspecific drug retention, we can conclude that the *unbound* plasma concentration (up) and the cellular concentrations (c), as well as the *total* plasma concentration (p) and the cellular concentrations (c) are proportional/linearly correlated.

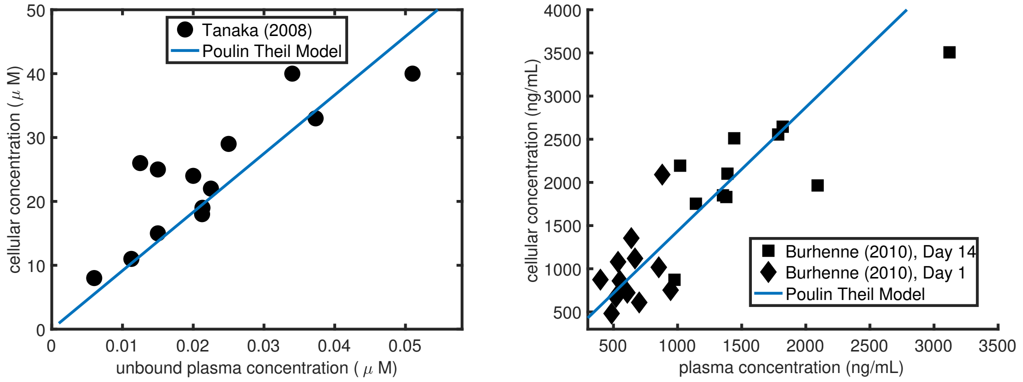

Figure S1.1: Relation between unbound plasma and intracellular EFV concentrations. **Left:** Solid black dots denote measured EFV concentrations in peripheral mononuclear blood cells vs. *unbound* plasma concentrations [2]. The solid blue line denotes cellular concentrations predicted by the Poulin-Theil model [1], which assumes passive diffusion of unbound drug and unspecific retention of drug in the intracellular space. Specifically, the model is used to predict a cell-to-*unbound* plasma partition coefficient $K_{c:up}$ according to $K_{c:up} \approx V_{\mathrm{w:c}} + P_{o:w} \cdot V_{\mathrm{nl:c}} + (0.3 \cdot P_{o:w} + 0.7)V_{\mathrm{np:c}}$, where we set $\log P_{o:w} = 4.6$ [5] and $V_{\mathrm{w:c}} = 0.76$, $V_{\mathrm{nl:c}} \approx 0.02$ and $V_{\mathrm{np:c}} \approx 0.01$ [3] denote the fractional volume of neutral- and phospholipids. Subsequently, $K_{c:up}$ is multiplied with the measured unbound plasma concentration to predict the cellular concentrations. **Right:** Solid black diamonds and squares denote measured EFV concentrations in peripheral mononuclear blood cells vs. *total* plasma concentrations at day 1 (trough concentrations, pre-steady state) and day 14 (trough concentrations, steady state) after initiation of efavirenz-based therapy [4]. The solid blue line denotes cellular concentrations predicted by the Poulin-Theil model [1], where we predicted the cell-to-*total* plasma partition coefficient $K_{c:p}$ according to $K_{c:p} = \frac{K_{c:up}}{1/f_u \cdot V_{\mathrm{w:p}} + P_{o:w} \cdot V_{\mathrm{nl:p}} + (P_{o:w} \cdot 0.3 + 0.7)V_{\mathrm{np:p}}}$, with $V_{\mathrm{w:p}} = 0.945$, $V_{\mathrm{nl:p}} = 0.0035$ and $V_{\mathrm{np:p}} = 0.00225$ [3] and $f_u = 0.002$.

S1.2 Free drug hypothesis

We previously showed that in the case of passive diffusion, a linear correlation exists between the *unbound* plasma concentration (up) and the cellular concentrations (c), as well as between the *total* plasma concentration (p) and the cellular concentration (c). The proportionality is precisely given by the drug

partition coefficient, such that $D_c = K_{c:up} \cdot D_{up}$, where D_c, D_{up} and $K_{c:up}$ denote the cellular drug concentration, the *unbound* drug concentration and the cell-to-*unbound* plasma drug partition coefficient. Importantly, this proportionality can be exploited when modelling the drug's effects, i.e.

$$\eta(t) = \frac{D_c^m}{D_c^m + \mathrm{IC}_{50,c}^m} = \frac{(K_{c:up} \cdot D_{up})^m}{(K_{c:up} \cdot D_{up})^m + \mathrm{IC}_{50,c}^m} = \frac{D_{up}^m}{D_{up}^m + \left(\frac{\mathrm{IC}_{50,c}}{K_{c:up}}\right)^m} = \frac{D_p^m}{D_p^m + \left(\frac{\mathrm{IC}_{50,c}}{K_{c:up} \cdot f_u}\right)^m} = \frac{D_p^m}{D_p^m + \mathrm{IC}_{50,p}^m}, \tag{S1.1}$$

which means that modelling the effect as a function of the plasma concentrations D_p and a 50% inhibitory plasma concentration $\mathrm{IC}_{50,p}$ is identical to modelling the effect based on target cell concentrations D_c with corresponding 50% inhibitory concentration $\mathrm{IC}_{50,c}$. In the equation above, f_u denotes the unbound fraction of the drug in the blood plasma.

S1.3 Ratio of *total* concentrations can be explained by protein binding

A number of studies [6–8] reported a low ratio between the *total* EFV concentrations in semen and plasma (reported values: 3-9%). Despite the low concentration gradient between semen and plasma, Avery et al. [7] reported that the unbound EFV concentrations in both compartments are of comparable magnitude. Here, we want to explore whether protein binding alone may explain reports of low tissue-to-plasma ratios reported by many authors (summarised in [9]). If protein binding can explain these observations, it lends further support for passive diffusion/equilibrative transport and the 'free drug hypothesis'.
The fraction of *unbound* drug in the plasma is given by

$$f_u^p = \frac{Kd}{Kd + Pr^p} \tag{S1.2}$$

$$\Leftrightarrow \frac{Kd}{Pr^p} = \frac{fu^p}{1 - fu^p} \tag{S1.3}$$

where Kd denotes the dissociation constant and Pr^p is the concentration of plasma protein that binds the drug (e.g. albumin). According to [10] the concentration of albumin in human serum is approximately 48 g/L. In contrast, it is about 1g/L in human semen [11]. Thus, we have

$$f_u^s = \frac{Kd}{Kd + Pr^p/48} \tag{S1.4}$$

$$= \frac{Kd/Pr^p}{Kd/Pr^p + 1/48} \tag{S1.5}$$

where f_u^s is the *unbound* fraction is the seminal plasma. Using eq. (S1.3) we obtain

$$f_u^s = \frac{f_u^p}{(1 - f_u^p)\left(\frac{f_u^p}{1 - f_u^p} + \frac{1}{48}\right)} \tag{S1.6}$$

$$= \frac{48 \cdot f_u^p}{47 \cdot f_u^p + 1} \tag{S1.7}$$

From the definition of the fraction *unbound*,

$$D_{\text{unbound}} = D_{\text{total}} \cdot f_u \Leftrightarrow D_{\text{total}} = \frac{D_{\text{unbound}}}{f_u}, \tag{S1.8}$$

where D_{total} and D_{unbound} denote the total and *unbound* drug concentrations, we can now compute the ratio of total concentrations, as reported by [6,7]. Assuming passive diffusion (i.e. the unbound concentrations in the semen and plasma are identical) we have

$$\frac{D_{\text{total}}^s}{D_{\text{total}}^p} = \frac{f_u^p}{f_u^s} = \frac{47 \cdot fu^p + 1}{48} \approx 2\% \tag{S1.9}$$

where we used $f_u^p = 0.002$. This calculation shows that the apparently low ratio of total semen-to-plasma concentration can be solely explained by differences in plasma protein binding (actually differences in the concentration of plasma proteins). Likewise, the same calculation can be applied to test whether the

ratio of total concentrations between the cervicovaginal fluid and the plasma can be solely explained by protein binding in the plasma. These ratios have been reported to be 0.4% [12], 1% [13] and < 8% [14], respectively. Notably, the cervicovaginal fluid concentrations of albumin and other drug-binding plasma proteins is only about 1% of the serum concentrations [15, 16]. Substituting these numbers in the equations above yields ratios of ≈ 1.2%, which explains the experimental estimates solely on the basis of protein binding.

S1.4 Sensitivity with respect to uncertainties in f_u

Since efavirenz is highly bound ($f_u \leq 1\%$), it might be difficult to accurately determine f_u in practice. Since we use the parameter f_u to correct the *in vitro* measured 50% inhibitory concentrations (which only used 50% plasma serum) according to [17] (Supplementary Text S3 therein):

$$\mathrm{IC}_{50,p} = \mathrm{IC}_{50,assay} \cdot \frac{2}{f_u + 1}, \tag{S1.10}$$

we wanted to assess whether uncertainties in f_u may affect some of our model predictions. Published values are in the range $0.001 \leq f_u \leq 0.01$ [4, 7, 18–20]. As can be seen, the denominator in eq. (S1.10), $f_u + 1 \approx 1$ for any of the published values and hence

$$\mathrm{IC}_{50,p} \approx \mathrm{IC}_{50,assay} \cdot 2, \tag{S1.11}$$

independent of f_u. Thus, uncertainties in f_u values have minimal impact on $\mathrm{IC}_{50,p}$ and consequently minimal impact on any of our predictions.

References

[1] Max von Kleist and Wilhelm Huisinga. Physiologically based pharmacokinetic modelling: a sub-compartmentalized model of tissue distribution. *J Pharmacokinet Pharmacodyn*, 34:789–806, 2007.

[2] Rie Tanaka, Hideji Hanabusa, Ei Kinai, Naoki Hasegawa, Masayoshi Negishi, and Shingo Kato. Intracellular efavirenz levels in peripheral blood mononuclear cells from human immunodeficiency virus-infected individuals. *Antimicrob Agents Chemother*, 52(2):782–5, Feb 2008.

[3] Patrick Poulin and Frank-Peter Theil. Prediction of pharmacokinetics prior to in vivo studies. 1. mechanism-based prediction of volume of distribution. *J Pharm Sci*, 91(1):129–56, Jan 2002.

[4] Jürgen Burhenne, Anne-Kathrin Matthée, Ivana Pasáková, Claudia Röder, Tilman Heinrich, Walter Emil Haefeli, Gerd Mikus, and Johanna Weiss. No evidence for induction of abc transporters in peripheral blood mononuclear cells in humans after 14 days of efavirenz treatment. *Antimicrob Agents Chemother*, 54(10):4185–91, Oct 2010.

[5] Drugbank entry for efavirenz (`https://www.drugbank.ca/drugs/DB00625`, accessed 13-aug-2018).

[6] Y Sunila Reddy, S Karl Gotzkowsky, Joseph J. Eron, Julie Y. Kim, William D. Fiske, Susan A. Fiscus, Leslie Petch, Myron S. Cohen, and Angela D M. Kashuba. Pharmacokinetic and pharmacodynamic investigation of efavirenz in the semen and blood of human immunodeficiency virus type 1-infected men. *J Infect Dis*, 186(9):1339–1343, Nov 2002.

[7] L B Avery, R P Bakshi, Y J Cao, and C W Hendrix. The male genital tract is not a pharmacological sanctuary from efavirenz. *Clin Pharmacol Ther*, 90:151–156, 2011.

[8] S Taylor, H Reynolds, C A Sabin, S M Drake, D J White, D J Back, and D Pillay. Penetration of efavirenz into the male genital tract: drug concentrations and antiviral activity in semen and blood of HIV-1-infected men. *AIDS*, 15(15):2051–3, Oct 2001.

[9] Laura J Else, Stephen Taylor, David J Back, and Saye H Khoo. Pharmacokinetics of antiretroviral drugs in anatomical sanctuary sites: the male and female genital tract. *Antivir Ther*, 16:1149–1167, 2011.

[10] Arne T Høstmark, Sissel E Tomten, and John E Berg. Serum albumin and blood pressure: a population-based, cross-sectional study. *J Hypertens*, 23(4):725–30, Apr 2005.

[11] S Elzanaty, J Erenpreiss, and C Becker. Seminal plasma albumin: origin and relation to the male reproductive parameters. *Andrologia*, 39(2):60–5, Apr 2007.

[12] Julie B. Dumond, Rosa F. Yeh, Kristine B. Patterson, Amanda H. Corbett, Byung Hwa Jung, Naser L. Rezk, Arlene S. Bridges, Paul W. Stewart, Myron S. Cohen, and Angela D M. Kashuba. Antiretroviral drug exposure in the female genital tract: implications for oral pre- and post-exposure prophylaxis. *AIDS*, 21(14):1899–1907, Sep 2007.

[13] Awewura Kwara, Allison Delong, Naser Rezk, Joseph Hogan, Heather Burtwell, Stacy Chapman, Carla C Moreira, Jaclyn Kurpewski, Jessica Ingersoll, Angela M Caliendo, Angela Kashuba, and Susan Cu-Uvin. Antiretroviral drug concentrations and HIV RNA in the genital tract of HIV-infected women receiving long-term highly active antiretroviral therapy. *Clin Infect Dis*, 46(5):719–25, Mar 2008.

[14] Sherene S Min, Amanda H Corbett, Naser Rezk, Susan Cu-Uvin, Susan A Fiscus, Leslie Petch, Myron S Cohen, and Angela D M Kashuba. Protease inhibitor and nonnucleoside reverse transcriptase inhibitor concentrations in the genital tract of HIV-1-infected women. *J Acquir Immune Defic Syndr*, 37(5):1577–80, Dec 2004.

[15] I G Salas-Herrera, P Turner, and R M Pearson. Secretion of drugs into the human female genital tract. *Postgrad Med J*, 67(790):710–2, Aug 1991.

[16] I G Salas Herrera, R M Pearson, and P Turner. Quantitation of albumin and alpha-1-acid glycoprotein in human cervical mucus. *Hum Exp Toxicol*, 10(2):137–9, Mar 1991.

[17] Sulav Duwal, Laura Dickinson, Saye Khoo, and Max von Kleist. Mechanistic framework predicts drug-class specific utility of antiretrovirals for hiv prophylaxis. *PLoS Comput Biol*, 15(1):e1006740, 2019.

[18] Lisa M Almond, Patrick G Hoggard, Damitha Edirisinghe, Saye H Khoo, and David J Back. Intracellular and plasma pharmacokinetics of efavirenz in HIV-infected individuals. *J Antimicrob Chemother*, 56(4):738–744, 2005.

[19] L B Avery, N Sacktor, J C McArthur, and C W Hendrix. Protein-free efavirenz concentrations in cerebrospinal fluid and blood plasma are equivalent: applying the law of mass action to predict protein-free drug concentration. *Antimicrob Agents Chemother*, 57(3):1409–14, Mar 2013.

[20] Aurélie Fayet, Alexandre Béguin, Begona Martinez de Tejada, Sara Colombo, Matthias Cavassini, Stefan Gerber, Chin B Eap, Amalio Telenti, Thierry Buclin, Jérôme Biollaz, and Laurent A Decosterd. Determination of unbound antiretroviral drug concentrations by a modified ultrafiltration method reveals high variability in the free fraction. *Ther Drug Monit*, 30(4):511–22, Aug 2008.
