# Supplementary material for: The Utility of Efavirenz-based Prophylaxis Against HIV Infection. A Systems Pharmacological Analysis
Source: Front Pharmacol. 2019 Mar 13;10:199. doi: 10.3389/fphar.2019.00199 (PMC6424904; doi:10.3389/fphar.2019.00199)
Supplement: Supplementary Text 2 — This supplementary text quantifies the probability of EFV resistance emergence prior to PEP initiation. We derive the probability of resistance emergence as a function of the time between virus exposure and start of post-exposure prophylaxis. The aim is to provide decision support on whether to start PEP after suspected exposure or not. [file Data_Sheet_4.PDF]

Supplementary Text S2.

In this supplementary material we compute the probability that any of the three resistance mutants G190S, K103N, Y181C emerges in the time between viral exposure and PEP initiation. As can be seen in Figure S2.1 the risk of resistance emergence increases with increasing delay time, contraindicating delayed PEP initiation.

S2.1 Probability of resistance mutation

The probability that any one of the three resistance mutations G190S, K103N and Y181C occurs, was computed based on the procedures and values from the supplementary material of [1].

For each of the resistance mutations where amino acid A changes to B, the probability of a mutation via a single-nucleotide change can be computed as outlined below: The amino acid A can be encoded by different nucleotide triplets due to the degenerated genetic code, i.e. if amino acid A is observed, there is a probability $P(\mathrm{XYZ}|\mathrm{A})$ that A is encoded by XYZ. Therefore, for each codon XYZ that encodes the amino acid A, denoted here as $a(\mathrm{XYZ}) = \mathrm{A}$, all single-nucleotide changes need to be examined that encode amino acid B: For all codons X'YZ, XY'Z or XYZ' where $a(\mathrm{X'YZ}) = \mathrm{B}$, $a(\mathrm{XY'Z}) = \mathrm{B}$ or $a(\mathrm{XYZ'}) = \mathrm{B}$, the probabilities need to be summed up and multiplied by the probability of A being encoded by XYZ:

$$P(\mathrm{A} \to \mathrm{B}) = \sum_{\mathrm{XYZ}:\, a(\mathrm{XYZ}) = \mathrm{A}} P(\mathrm{XYZ}|\mathrm{A}) \cdot \left(\sum_{\mathrm{X'}:\, a(\mathrm{X'YZ}) = \mathrm{B}} P(\mathrm{XYZ} \to \mathrm{X'YZ}) + \sum_{\mathrm{Y'}:\, a(\mathrm{XY'Z}) = \mathrm{B}} P(\mathrm{XYZ} \to \mathrm{XY'Z}) + \sum_{\mathrm{Z'}:\, a(\mathrm{XYZ'}) = \mathrm{B}} P(\mathrm{XYZ} \to \mathrm{XYZ'}) \right) \quad \text{(S2.1)}$$

where $\to$ denotes "mutates to". Assuming statistical independence, the probability that the corresponding codon is XYZ when amino acid A is observed, is given by:

$$P(\mathrm{XYZ}|\mathrm{A}) = \frac{\text{frequency of XYZ}}{\text{total frequency of all codons } \mathrm{C_1C_2C_3} \text{ that encode A}} = \frac{f(\mathrm{X}) \cdot f(\mathrm{Y}) \cdot f(\mathrm{Z})}{\sum_{\mathrm{C_1C_2C_3}\,:\, a(\mathrm{C_1C_2C_3}) = \mathrm{A}} f(\mathrm{C_1}) \cdot f(\mathrm{C_2}) \cdot f(\mathrm{C_3})} \quad \text{(S2.2)}$$

where $f(\mathrm{X})$ is the frequency of nucleotide X in the HIV genome. Together with the probabilities of a single-nucleotide change, these values are listed in Table S2.1.

Nucleotide	Mutation rate				Frequency
	U	C	A	G	
U	-	$1.1 \cdot 10^{-5}$	$1.4 \cdot 10^{-5}$	$3.7 \cdot 10^{-6}$	0.223
C	$2.5 \cdot 10^{-5}$	-	$6.7 \cdot 10^{-6}$	$1.8 \cdot 10^{-7}$	0.182
A	$8.2 \cdot 10^{-7}$	$5.5 \cdot 10^{-7}$	-	$1.2 \cdot 10^{-5}$	0.351
G	$8.8 \cdot 10^{-7}$	$8.8 \cdot 10^{-7}$	$5.7 \cdot 10^{-5}$	-	0.244

Table S2.1: Mutation rates and nucleotide frequencies in the HIV genome. The mutation rates are given per base and per HIV reverse transcription process. All values were taken from the supplementary material of [1].

Using this computation, we obtain $P_{\mathrm{RT}}(\mathrm{G190S}) = 2.3085 \cdot 10^{-5}$, $P_{\mathrm{RT}}(\mathrm{K103N}) = 1.5301 \cdot 10^{-6}$ and $P_{\mathrm{RT}}(\mathrm{Y181C}) = 1.2 \cdot 10^{-5}$ meaning the total mutation probability per reverse transcription event for these three mutations is $\mu = 3.66151 \cdot 10^{-5}$.

S2.2 Probability of resistance emergence before PEP initiation

To assess the probability of resistance emergence before PEP initiation, we performed stochastic simulations and counted how many times the reverse transcription process occurred before the start of PEP. We

denote this number as N_{RT}.

We then computed the probability that at least one of the three resistant mutations emerged during this waiting period as

$$P(\text{at least one resistance mutation emerges}) = 1 - P(\text{no resistance mutation emerges}) = 1 - (1-\mu)^{N_{\mathrm{RT}}(t_{\mathrm{delay}})} \tag{S2.3}$$

Figure S2.1 displays the probability of resistance emergence for those three mutants (dashed red line). The graphic shows that when PEP is started within 48h after the viral exposure, the probability of resistance emergence is $\leq 0.14\%$ and therefore negligible. However, when PEP starts 72h after exposure or later, this probability surpasses 1% and approximates the probability of infection in the absence of antivirals (ca. 3%, compare with the dotted blue line). This means that either the virus went extinct or developed resistance with 38% probability. Consequently, PEP is likely to fail. Initiating PEP 96h after exposure results in a probability of resistance emergence of $\approx 2\%$, meaning $\approx 70\%$ of individuals where the virus did not go extinct before the start of PEP will have acquired resistances.

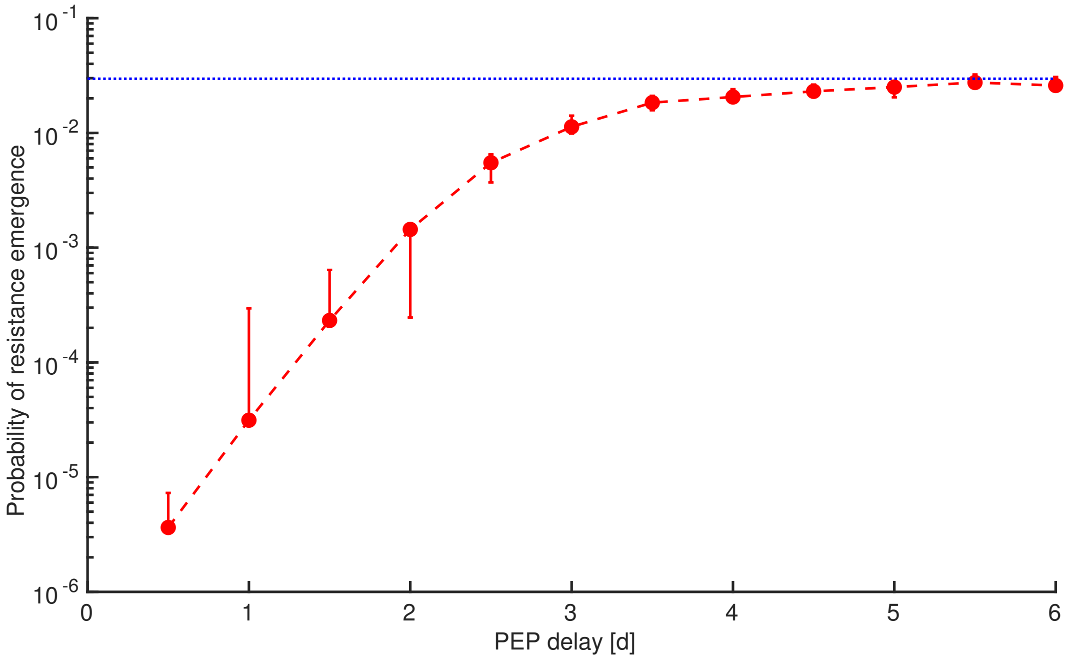

Figure S2.1: Probability of resistance emergence before PEP initiation. The probability of resistance emergence was computed from stochastic simulations of the viral dynamics model. The dashed red line shows the probabilities per PEP delay scenario and the error bars were computed using Greenwood's formula. The dotted blue line shows the probability of infection in the absence of any antivirals.

References

[1] Ameya R Kirtane, Omar Abouzid, Daniel Minahan, Taylor Bensel, Alison L Hill, Christian Selinger, Anna Bershteyn, Morgan Craig, Shirley S Mo, Hormoz Mazdiyasni, Cody Cleveland, Jaimie Rogner, Young-Ah Lucy Lee, Lucas Booth, Farhad Javid, Sarah J Wu, Tyler Grant, Andrew M Bellinger, Boris Nikolic, Alison Hayward, Lowell Wood, Philip A Eckhoff, Martin A Nowak, Robert Langer, and Giovanni Traverso. Development of an oral once-weekly drug delivery system for HIV antiretroviral therapy. *Nat Commun*, 9(1):2, 01 2018.
