# Supplementary material for: The Utility of Efavirenz-based Prophylaxis Against HIV Infection. A Systems Pharmacological Analysis
Source: Front Pharmacol. 2019 Mar 13;10:199. doi: 10.3389/fphar.2019.00199 (PMC6424904; doi:10.3389/fphar.2019.00199)
Supplement: Supplementary Table 1 — The table shows the individual pharmacokinetic parameters (CLss/Fbio, V/Fbio and ka) of all virtual patients. [file Data_Sheet_2.PDF]

Table S1 Composite genotype subgroups and corresponding relative changes in efavirenz apparent oral clearance (estimate and relative standard errors) compared to the reference genotype as determined by the final population PK model. Table reproduced from Dickinson et al. (2015) *Clin Pharmacol Ther* 98(4):406–416.

***CYP2B6* 516G>T/983T>C/*CYP2A6**9B/*17 combinations**	**Estimate[*]**	**RSE (%)**
GG/TC or CC/CC/CC	0.73 (8.7)	18.4
GG/TC or CC/CC/CT or TT	0.30 (3.5)	20.6
GG/TC or CC/CA or AA/CC	0.75 (9.0)	32.8
GG/TC or CC/CA or AA/CT	0.96 (11.4)	2.4
GT/TT/CC/CC	0.75 (8.9)	3.7
GT/TT/CC/CT or TT	0.68 (8.1)	8.8
GT/TT/CA or AA/CC	0.69 (8.2)	5.7
GT/TT/CA or AA/CT	0.71 (8.4)	5·5
GT/TC or CC/CC/CC	0.42 (5.0)	24.5
GT/TC or CC/CC/CT or TT	0.39 (4.6)	30.1
GT/TC or CC/CA or AA/CC	0.18 (2.2)	18.1
TT/TT/CC/CC	0.33 (4.0)	6.6
TT/TT/CC/CT or TT	0.34 (4.1)	19.6
TT/TT/CA or AA/CC	0.44 (5.2)	25.0
TT/TT/CA or AA/CT	0.75 (8.9)	74.1
Missing genotype	0.73 (8.7)	8.3

[*] Covariate estimate: relative change in *CYP2B6* 516G>T/983T>C/*CYP2A6**9B/*17 composite genotype compared to reference (GG/TT/CC/CC, GG/TT/CC/CT or TT, GG/TT/CA or AA/CC, GG/TT/CA or AA/CT or TT) with corresponding typical population clearance value for efavirenz (L/h) in brackets for a patient weighing 70kg with a particular genotype

RSE: relative standard error; SE: standard error

$RSE = (SE_{ESTIMATE}/ESTIMATE) * 100$
